# Type I Choledochal Cyst in a Three-Year-Old with Recurrent Vomiting and Pancreatitis: A Case Report

**DOI:** 10.5811/cpcem.63654

**Published:** 2026-10-07

**Authors:** Dominic DiPrinzio, Rance Del Mar

**Affiliations:** * University of Nevada, School of Medicine, Renown Regional Medical Center, Department of Emergency Medicine, Reno, Nevada; † University of Nevada, School of Medicine, Reno, Nevada

**Keywords:** choledochal cyst, pancreatitis, pediatrics, abdominal pain, case report

## Abstract

**Introduction:**

Choledochal cysts are rare congenital dilatations of the biliary tree that may present with nonspecific gastrointestinal symptoms in children, posing diagnostic challenges.

**Case Report:**

We report a 3-year-old girl who presented with recurrent vomiting and abdominal pain. Initial laboratory testing revealed a mildly elevated lipase. An abdominal ultrasound demonstrated a pancreatic cyst; the patient was discharged with outpatient follow-up. She returned with worsening symptoms and a markedly elevated lipase of 2,247 U/L (reference range, < 160 U/L). Computed tomography subsequently revealed a type I choledochal cyst. She underwent operative resection with an uncomplicated postoperative course.

**Conclusion:**

Persistent gastrointestinal symptoms with uptrending lipase in children should prompt consideration of hepatobiliary etiologies. Choledochal cysts may be missed on initial presentation as the classic triad of jaundice, palpable abdominal mass, and right upper quadrant pain is present in a minority of cases.

## INTRODUCTION

Choledochal cysts are rare congenital anomalies of the biliary tract with variable presentations, accounting for approximately one in 100,000 to 150,000 live births in Western populations.^1^ Clinical symptoms are frequently nonspecific in children, contributing to delayed diagnosis and potential complications such as recurrent pancreatitis, cholangitis, hepatic fibrosis, and malignancy.^2^,^3^ We present a case of a 3-year-old girl whose evolving symptoms led to the eventual identification of a type I choledochal cyst requiring definitive surgical intervention.

## CASE REPORT

A previously healthy 3-year-old girl presented to a community emergency department (ED) with two episodes of nonbloody, nonbilious vomiting that were preceded by brief episodes of abdominal pain, which started after she struck her abdomen on a bar of a play structure in a park. The pain resolved after vomiting. There were no associated fevers. Vital signs were as follows: blood pressure, 104/57 mm Hg; pulse, 91 beats per minute; temperature, 36.1 °C; respiratory rate, 26 breaths per minute; and weight, 18.8 kg. The physical examination revealed no abdominal discomfort on deep palpation, and no focal tenderness or palpable masses. Abdominal radiograph revealed a nonobstructive bowel gas pattern. She tolerated oral hydration and was discharged with supportive care instructions.

The patient returned four hours later after another episode of vomiting at home. Abdominal examination was remarkable for right lower quadrant tenderness. Laboratory testing noted a lipase of 182 U/L (reference range, < 160 U/L), and normal complete blood count, C-reactive protein, and comprehensive metabolic panel. Abdominal ultrasound demonstrated a normal appendix and a 2 × 3 cm-anechoic pancreatic cyst; no biliary dilation was documented (Image 1). The patient was given ondansetron, after which she was able to tolerate oral liquids and had no further episodes of vomiting or pain. She was discharged with a bland diet and close outpatient follow-up.

Over the next two days, the patient improved clinically while adhering to a bland diet. However, when her diet was advanced, vomiting recurred and was accompanied by right lower quadrant pain. She re-presented to the ED, where examination showed abdominal tenderness in the epigastric and right lower quadrant region. Repeat laboratory evaluation revealed a lipase of 2,247 U/L. Computed tomography (CT) of the abdomen and pelvis demonstrated a cystic structure measuring 6 cm in its greatest dimension, extending from the pancreatic head into the porta hepatis, which was concerning for a choledochal cyst (Image 2).

In addition, there was notable thickening of the terminal ileum with associated prominent right lower quadrant lymphadenopathy and a normal appendix. She was admitted and underwent magnetic resonance cholangiopancreatography. Her pancreatitis was managed, and she was discharged home after three days. Her surgery was scheduled two days after discharge to allow additional time for her lipase to return to normal. She then underwent robotic choledochal cystectomy with a primary hepaticoduodenostomy. Her postoperative recovery was uneventful, and she was discharged home on post operative day 4 with surgical follow-up. At the time of follow-up, she remained asymptomatic with normal diet tolerance.

## DISCUSSION

Choledochal cysts are classified into five primary types using the Todani system.^4^ Type I choledochal cysts are a cystic or fusiform dilation of the extrahepatic common biliary duct.^4^ They are also the most common type, accounting for 50–80% of all cases.^4^ Other classifications include type II, which presents as a saccular diverticulum of the common bile duct, type III as a dilation of the distal portion of the common bile duct into the duodenal wall, and type IV as either intrahepatic and extrahepatic dilation of the biliary ducts or multiple sites of dilation in only the extrahepatic biliary tree.^4^ Type V choledochal cysts, known as Caroli disease, are characterized by multiple dilations of the intrahepatic bile ducts.^4^

*CPC-EM Capsule*

What do we already know about this clinical entity?

*Choledochal cysts are rare biliary dilatations causing obstruction, cholangitis, pancreatitis, and malignancy risk.*

What makes this presentation of disease reportable?

*Symptoms followed minor abdominal trauma with normal initial laboratory studies. Diagnosis was delayed until symptom progression prompted repeat evaluation.*

What is the major learning point?

*Consider choledochal cysts in recurrent biliary symptoms despite negative initial workup. Repeat assessment and additional imaging may reveal the diagnosis.*

How might this improve emergency medicine practice?

*Promotes broader differentials, repeat evaluation of persistent symptoms, earlier diagnosis, and timely specialist referral to prevent complications.*

Clinical manifestations vary by age, often contributing to a delay in diagnosis.^2^,^4^,^5^ Prenatal choledochal cysts can be identified through the presence of infrahepatic lesions, while infants (up to 12 months of age) present with jaundice, hepatomegaly, and acholic stools.^2^,^3^ Children and adolescents (up to 18 years ) account for nearly 80% of choledochal cyst cases and typically experience generic symptoms such as vomiting and abdominal pain or show evidence of pancreatitis.^2^,^5^,^6^ In a recent study, all three symptoms of the classic triad (right upper quadrant abdominal pain, jaundice, and a palpable abdominal mass) were observed in only 4% of pediatric patients.^6^ Thus, it is possible for patients to initially be discharged with nonspecific abdominal pain. In these cases, a return visit to the ED often serves as a critical diagnostic inflection point, signaling an evolving pathology and prompting a significant increase in imaging, laboratory tests, and procedures.^7^

It is hypothesized that abnormalities at the pancreaticobiliary duct junction result in reflux of pancreatic enzymes that weakens the biliary walls, contributing to cyst formation and pancreatitis.^1^ Elevations in young children’s pancreatic enzymes reflect recurrent chronic inflammation and are associated with precancerous changes within the biliary tract.^8^ Given the severity of these underlying pathologies, prompt diagnostic evaluation and early surgical intervention are essential.^3^ Ultrasound is a highly sensitive modality for the initial diagnosis of choledochal cysts (71–97%) and can be used to identify them prenatally.^4^ However, ultrasound is limited in its ability to detect subtle biliary dilation or definitively map biliary anatomy to identify Todani subtypes.^4^,^5^ Imaging such as CT with contrast or magnetic resonance cholangiopancreatography offer improved sensitivities and a more conclusive diagnosis.^4^,^5^

Surgical excision with biliary enteric reconstruction is the standard of care due to risks of recurrent pancreatitis, cholangitis, hepatic fibrosis, and malignancy if left untreated, which have been documented even during childhood.^2^,^9^ Minimally invasive techniques, including laparoscopic and robot-assisted approaches, continue to expand and demonstrate favorable outcomes in pediatric patients.^10^

Although choledochal cysts are well documented in the surgery literature, a review of prior emergency medicine case reports demonstrates that they remain a rare and difficult diagnosis in the ED. Initial imaging can mistakenly identify cysts as more common acute biliary pathology such as a distended gallbladder with shadowing stones, requiring additional scanning.^11^ Furthermore, rare complications such as spontaneous rupture can complicate the presentation and are difficult to diagnose preoperatively.^12^

This case emphasizes the importance of reconsidering differential diagnoses when symptoms evolve, particularly in pediatric patients with recurrent vomiting and rising pancreatic enzymes. Emergency clinicians should maintain a high index of suspicion for hepatobiliary pathology when initial imaging is nondiagnostic and clinical symptoms progress.

## CONCLUSION

Choledochal cysts may present subtly in children and can be initially missed when symptoms mimic common gastrointestinal illnesses. Worsening abdominal symptoms and elevated lipase levels should prompt further imaging and consideration of hepatobiliary disease. Early recognition and surgical management are essential to prevent complications.

## Figures and Tables

**Image 1:**
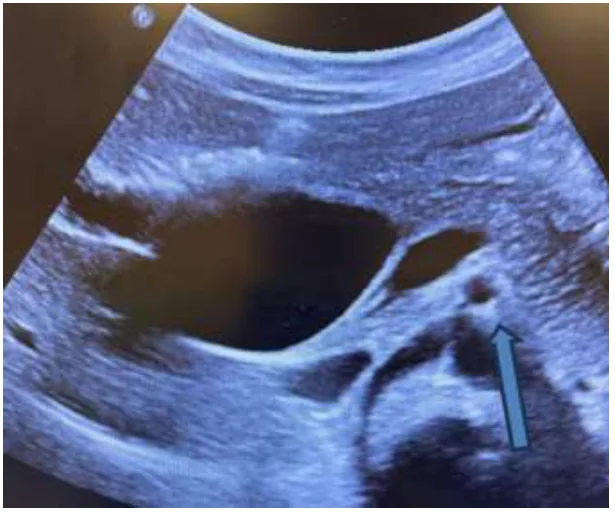
2 × 3 cm-cyst adjacent to gallbladder and pancreas (arrow).

**Image 2:**
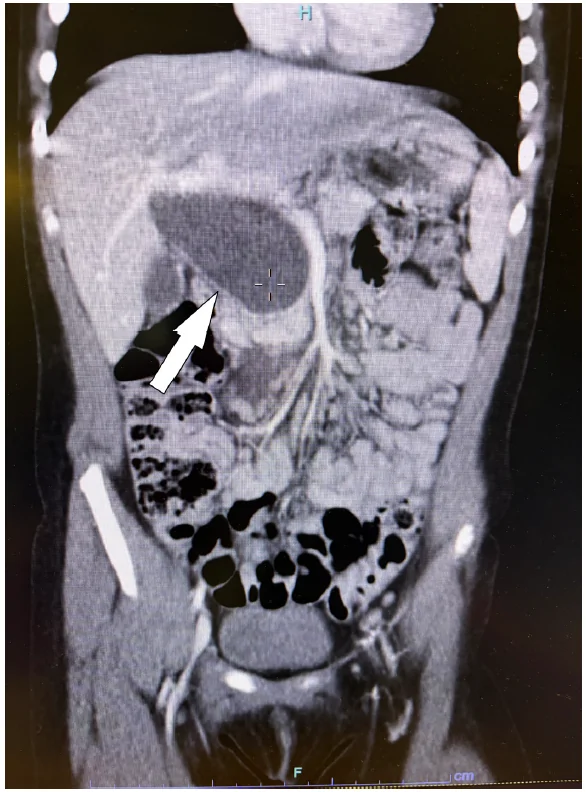
Coronal computed tomography of the abdomen and pelvis demonstrating cystic structure (white arrow) extending from the pancreatic head into the porta hepatis, concerning for a type I choledochal cyst.
